# Effects of *Lonicera japonica* Flower Bud Extract on *Citrobacter rodentium*-Induced Digestive Tract Infection

**DOI:** 10.3390/medicines7090052

**Published:** 2020-08-27

**Authors:** Masaaki Minami, Toshiaki Makino

**Affiliations:** 1 Department of Bacteriology, Graduate School of Medical Sciences, Nagoya City University, 1 Kawasumi, Mizuho-ku, Nagoya 467-8601, Japan; 2 Department of Pharmacognosy, Graduate School of Pharmaceutical Sciences, Nagoya City University, 3-1 Tanabe-Dori, Mizuho-ku, Nagoya 467-8603, Japan; makino@phar.nagoya-cu.ac.jp

**Keywords:** *Lonicera japonica*, *Citrobacter rodentium*, bacterial digestive tract infection, chlorogenic acid, intraperitoneal macrophages

## Abstract

**Background:** Although antibiotic therapy is currently a gold standard for bacterial infections, it is not used for severe diseases like enterohemorrhagic *Escherichia coli*, in which the Shiga toxin is overproduced by antibiotic action. The *Lonicera japonica* flower bud (LJF) is an herbal component used against purulent diseases in traditional Japanese and Chinese medicine. We investigated the effects of LJF extract (LJFE) on *Citrobacter rodentium*-induced digestive tract infection in a mouse model. **Methods:** *Citrobacter rodentium* and LJFE were orally administered to C57BL/6 mice. The survival rate and bacterial colonization in the large intestine, mesenteric lymph node, and blood of mice were evaluated. Cytokines secreted from intraperitoneal macrophages of LJFE-treated mice were measured using ELISA. Moreover, the phagocytic activity of intraperitoneal macrophages against *Citrobacter rodentium* was compared between LJFE- or chlorogenic acid (CGA)-treated mice. **Results:** LJFE significantly increased the survival rate and decreased *Citrobacter rodentium* colonization in mice. Moreover, the values of tumor necrosis factor-α, interleukin-1β, and interferon-γ secreted from macrophages were increased following LJFE treatment. While macrophages of LJFE-treated mice showed a significant phagocytic activity, macrophages of CGA-treated mice only showed a phagocytic tendency. **Conclusions:** LJF may be useful for treating *Citrobacter rodentium*-induced digestive tract infection.

## 1. Introduction

Bacterial infections of the gastrointestinal tract, including pathogenic *Escherichia coli* O157 infections, are important communicable diseases because they infect many people and include deaths [1,2]. Even though antibiotic treatment is the gold standard for bacterial infections, sensitivity to antibiotics has decreased due to the spread of multidrug-resistant bacteria, such as extended spectrum β-lactamase- and carbapenemase-producing bacteria [3,4]. Furthermore, excess production of several bacterial toxins, such as Shiga toxins, occurs as a result of antibiotic use against O157, which leads to hemolytic uremic syndrome [5]. However, the use of antibiotic therapy is avoided for some diseases. Thus, it is necessary to develop new therapeutic methods that do not rely on antibiotics.

*Lonicera japonica* is one of the popular medicinal plants among honeysuckles, and its dried flower bud (LJF) is used as an antipyretic and detoxification agent in traditional Chinese medicine (TCM) [6]. In TCM, LJF is used as a detoxification agent for purulent diseases and an anti-inflammatory agent for upper respiratory tract inflammation (e.g., *wind fever*). In addition, LJF may be effective against pharyngitis and pneumonia, as respiratory infections might be the target of its broad medicinal effects [7]. Although digestive tract infection is considered to cause enteritis, most studies testing the impacts of LJF were performed in animal models of respiratory infections, such as pneumonia, and there are few studies on its effectiveness against purulent diseases, such as gastrointestinal tract infections [8].

Therefore, to clarify the effectiveness of LJF against diseases other than respiratory tract infections, we used a mouse model of *Citrobacter rodentium* infection in this study, which showed the same symptoms as human O157 digestive tract infectious disease [9]. The objective of this study was to prevent the spread of multidrug-resistant bacteria by establishing a novel treatment method for bacterial digestive tract infections using crude drugs without relying on antibiotics.

## 2. Materials and Methods

### 2.1. LJF Extract (LJFE) Preparation

**Figure 1:**
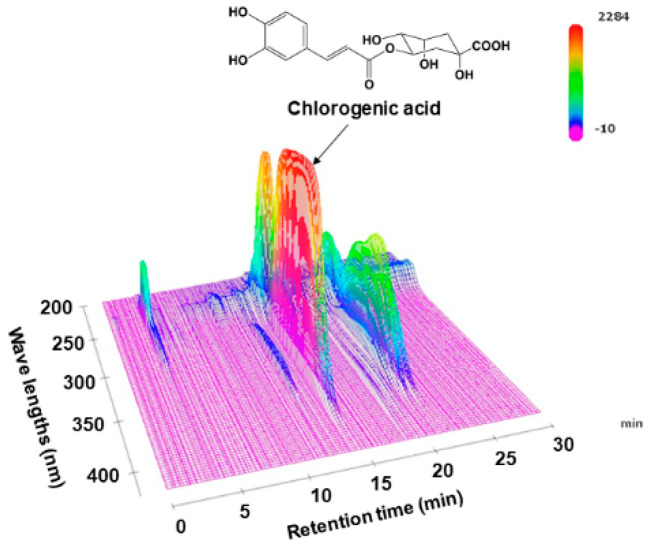
Three-dimensional high-performance liquid chromatogram of *Lonicera japonica* flower bud extract (LJFE).

Dried LJFs (lot number, 9I15) were purchased from Daiko Shoyaku (Nagoya, Japan). Dried LJFs (25 g) were boiled in water (600 mL) for 30 min, filtrated, and lyophilized to obtain the extract (LJFE) (9.2 g, 36.8%). For the fingerprint analysis, we dissolved the extract (25 mg) in MeOH (0.5 mL). After centrifuging (15,000 rpm for 5 min), we subjected the supernatant (4 μL) to HPLC. The description of the system is as follows: Shimadzu LC-10A_VP_ (Kyoto, Japan); column, TSK-GEL ODS-80TS (4.6 mm × 250 mm; Tosoh, Tokyo, Japan); mobile phase, 0.5% HCOOH in H_2_O/0.5% HCOOH in CH_3_CN 97:3 (0 min)–0:100 (60 min), linear gradient; flow rate, 1.0 mL/min; column temperature, 40 °C; and detection wave length, 200–600 nm (using a photodiode array detector). Chromatograms at 0–30 min and 200–420 nm were presented (Figure 1), without any peaks in these ranges. Chlorogenic acid (CGA) (Sigma–Aldrich, St. Louis, MO, USA) was identified based on the retention time (14.4 min) and UV spectrum of the standard compound. The linear regression of the CGA concentration range (0.5–5 µg) was calibrated based on the peak area detected at 340 nm by utilizing the least-squares method (*r*^2^ > 0.999).

### 2.2. Bacteria

*Citrobacter rodentium* (ATCC51459) DSB100 (ATCC, Manassas, VA, USA) was purchased. Bacteria were cultured on LB agar medium (Becton Dickinson and Company, Franklin Lakes, NJ, USA) for 24 h at 37 °C. To examine the in vitro antibacterial effects of LJFE, *Citrobacter rodentium* (1 × 10^6^ CFU) was cultured in liquid LB medium (Becton Dickinson and Company) containing LJFE (final concentration 0–5 mg/mL) at 37 °C for 24 h. The bacterial growth was measured with a turbidity meter (O.D. 600).

### 2.3. Animal Study

In the infection experiment, female C57BL/6 mice (six weeks old) (Japan SLC Inc., Hamamatsu, Japan) were used. One group consisted of eight mice. To examine the in vivo antibacterial effect of LJFE, *Citrobacter rodentium* was cultured on LB agar medium (Becton Dickinson and Company) at 37 °C for 24 h on the day before the infection. On the day of the infection, the bacteria were suspended in sterile PBS, and mice in each group were orally administered 1 × 10^8^ CFU bacteria under anesthesia. We defined the viable bacterial count of the inoculum by serial dilution and plating on LB agar. Mice were orally administrated polymyxin B sulfate (100 mg/kg/day; Fujifilm Wako Pure Chemical Co., Osaka, Japan) on the day before the infection because the administration of polymyxin B reduces the number of indigenous bacteria in the intestinal tract, thereby facilitating the detection of Citrobacter [10]. The maximum dose of LJF is described as 15 g/day in Chinese herbal medicine [11]. Using the ratio of yielded extract described above and the body weight, this dose corresponds to LJFE 0.11 g/kg/day. Usually, the dose for animal experiments is approximately 10–20-fold the human dosage; we chose a LJFE dose of 1 or 2 g/kg/day. LJFE was orally administrated in physiological saline or the vehicle once a day using a gastric tube from the day before the infection until the end of the observation. To evaluate the therapeutic effects, the general condition of the mice was observed over time. When an infected mouse died or seven days after the infection, mice were euthanized by CO_2_, and their tissues were collected. A part of the large intestine was fixed in formalin, and a histological analysis was carried out utilizing hematoxylin and eosin (H & E) staining. Another part of the large intestine, and the mesenteric lymph node of the mice were homogenized in saline. Blood was also aseptically separated from the mice. Aliquots of blood and the homogenates were serially diluted and cultured on MacConkey agar medium (Eiken Chemical Co., Ltd., Tokyo, Japan) for 24 h at 37 °C. Then, we counted the number of bacterial colonies. All animal experiments were approved by Nagoya City University Committee (Ethical approval code: H28M-05; Date of approval: 2 March 2016).

### 2.4. Analysis of Ex Vivo Effects of LJFE on Immune Functions

Ex Vivo animal studies were performed with some modifications [12]. Serum and intraperitoneal macrophages were collected from mice treated with oral LJFE (2 g/kg body weight), CGA (9.2 mg/kg body weight), or saline once a day for seven days. After the peritoneal macrophage cells were harvested by lavage with cold sterilized PBS from the peritoneal cavities, they were cultured at 37 °C with 5% CO_2_ atmosphere for 24 h. Intraperitoneal macrophages (1 × 10^6^ cells) and *Citrobacter rodentium* (1 × 10^5^ CFU) opsonized in advance with mouse serum were added to RPMI1640 (Fujifilm Wako, Osaka, Japan) and cocultured at 37 °C for 60 min in the presence of 5% CO_2_. After 24 h, we counted the bacterial colonies found in the MacConkey agar culture with serial dilution. Intraperitoneal macrophages collected from the mice treated with LJFE were cultured in RPMI1640 supplemented with 5% fetal calf serum (FCS, Sigma–Aldrich, St. Louis, MO, USA) for 24 h at 10^6^ cells/mL. We measured the values of interleukin (IL)-1β, tumor necrosis factor (TNF)-α, interferon (IFN)-γ, and IL-6 in the culture supernatant by utilizing ELISA kits (BioLegend, San Diego, CA, USA). In another experiment, 10^6^ cells/mL intraperitoneal macrophages were cultured in RPMI1640 containing 5% FCS and ^3^H-thymidine (2.0 Ci/mL, PerkinElmer, Inc., Waltham, MA, USA) for 24 h. We measured the radioactivity of the cells by utilizing a liquid scintillation counter.

### 2.5. Statistical Analysis

We carried out statistical analyses by utilizing an unpaired *t* test for two groups, Turkey’s multiple comparison test with Bonferroni’s correction for three or more groups, and Kaplan–Meier log rank test with Bonferroni’s correction for the survival curves. A *p* < 0.05 was regarded as statistically significant.

## 3. Results

### 3.1. HPLC Chromatogram of LJFE

Before studying the pharmacology of LJFE, we analyzed LJFE chemically in order to certificate the reproducibility of the study. Figure 1 shows the fingerprint pattern of LJFE by HPLC-PDA analysis. The concentration of CGA, the marker compound, in LJFE was 0.46 (*w*/*w*, %).

### 3.2. In Vitro Effects of LJFE against Citrobacter rodentium

In this study, the direct antibacterial effects of LJFE on *Citrobacter rodentium* DSB100 were measured based on the turbidity of bacterial suspensions; however, there were no significant differences among the absorbance values of bacterial cultures with different LJFE concentrations. Therefore, LJFE produced no direct antibacterial effect against *Citrobacter rodentium*, even at the maximum concentration of 5 mg/mL (data not shown).

### 3.3. In Vivo Effects of LJFE against Citrobacter rodentium in a Mouse Model

**Figure 2:**
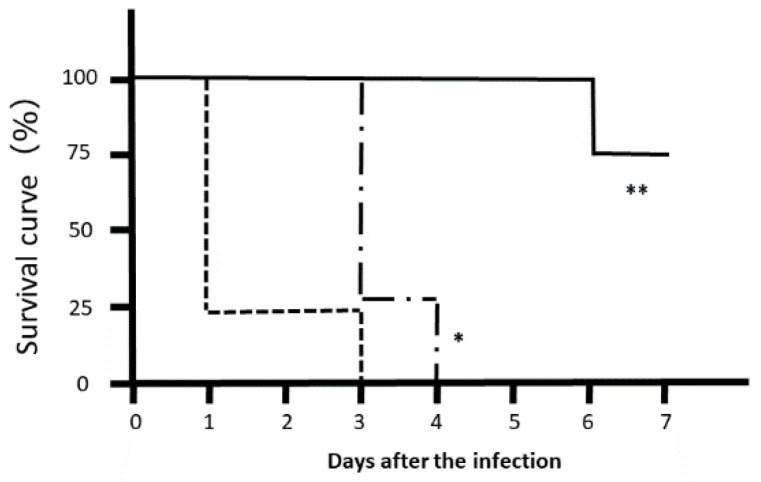
Effects of *Lonicera japonica* flower bud extract (LJFE) on the survival rate of *Citrobacter*-infected mice. Eight mice were orally infected with *Citrobacter*, and their survival rate was plotted. Dotted line, control group without LJFE treatment; dashed line: LJFE-treated group (1 g/kg); solid line, LJFE-treated group (2 g/kg). * *p* < 0.05, ** *p* < 0.01 compared with the control group (Kaplan–Meier log rank test).

**Figure 3:**
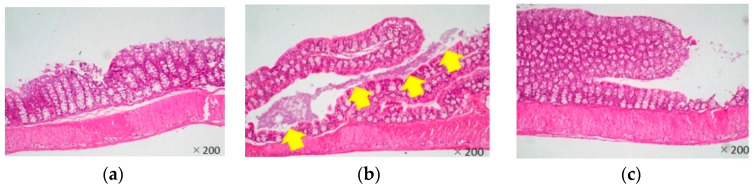
Effects of *Lonicera japonica* flower bud extract (LJFE) on the large intestine histology of *Citrobacter*-infected mice. Hematoxylin and eosin staining were performed. The yellow arrow points to the bacterial mass. (**a**) Normal mouse, (**b**) *Citrobacter*-infected control group without LJFE-treatment, (**c**) *Citrobacter*-infected group treated with LJFE (2 g/kg).

**Figure 4:**
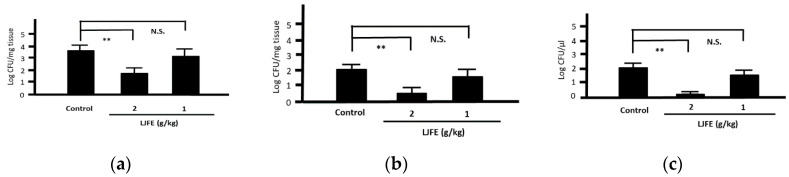
Effects of *Lonicera japonica* flower bud extract (LJFE) on bacterial colonization in *Citrobacter*-infected mice. Bacteria colonies in the (**a**) large intestine, (**b**) mesenteric lymph nodes, and (**c**) blood were represented. Data are presented as mean ± S.D. (*n* = 6). ** *p* < 0.01, N.S. not significant, Turkey’s multiple comparison test.

Most *Citrobacter rodentium*-infected mice died one day after the infection. However, the survival rates of LJFE-treated mice increased (until five days; *p* < 0.01) according to the dose (Figure 2). In the histopathological analysis, although a bacterial mass was observed in the colonic mucosa in bacterial-infected mice, it was not visible in the LJFE (2 g/kg LJFE group)-administered bacterial-infected mice (Figure 3). Moreover, the number of *Citrobacter rodentium* colonies in the large intestine, mesenteric lymph nodes, and blood in the LJFE-treated group was significantly depressed relative to that in the control group (*p* < 0.01) (Figure 4).

### 3.4. Effects of LJFE on Serum Cytokine Levels of Mice

**Figure 5:**
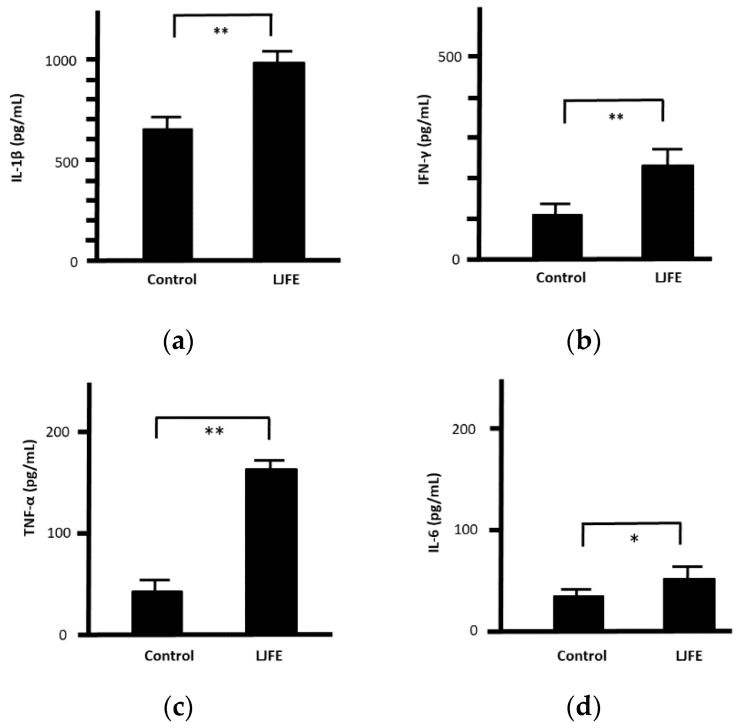
Effects of *Lonicera japonica* flower bud extract (LJFE) on mice serum cytokine levels. Mice were orally administered LJFE (2 g/kg) for seven days, and the serum levels interleukin (**a**) (IL)-1β, (**b**) interferon (IFN)-γ, (**c**) tumor necrosis factor (TNF)-α, and (**d**) IL-6 were measured by ELISA. Data are presented as mean ± S.D. (*n* = 6). * *p* < 0.05, ** *p* < 0.01, *t*-test.

Upon confirming its in vivo effects, we next examined the antibacterial effects of LJFE ex vivo. The serum values of IL-1β, INF-γ, TNF-α, and IL-6 in LJFE-treated mice (2 g/kg) were significantly higher (*p* < 0.01 or 0.05) (Figure 5).

### 3.5. Ex Vivo Effects of LJFE on the Phagocytic Activity of Peritoneal Macrophages against Citrobacter rodentium in Mice

**Figure 6:**
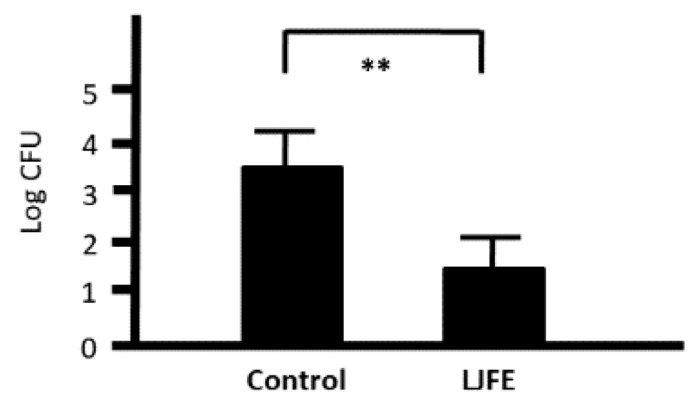
Ex Vivo effects of *Lonicera japonica* flower bud extract (LJFE) on the phagocytic activity of intraperitoneal macrophages against *Citrobacter* in mice. Mice were orally administered LJFE (2 g/kg) for seven days, and their peritoneal macrophages were cultured with *Citrobacter*. Bacterial colonies at 1 h after the start of phagocytosis were demonstrated. Data are presented as mean ± S.D. (*n* = 6). ** *p* < 0.01, *t*-test.

Next, we investigated the phagocytic activity of intraperitoneal macrophages as a measure of cell-mediated immunity. We cocultured 1 × 10^5^ CFU bacteria with 1 × 10^6^ peritoneal macrophages collected from both control and LJFE-treated mice. At 1 h after the start of phagocytosis, the bacterial number was decreased even in the control group, confirming the phagocytic activity of peritoneal macrophages. Nonetheless, the number of bacteria was significantly decreased in the LJFE-treated mice when compared with that in the control mice (*p* < 0.01) (Figure 6). This result indicates that LJFE treatment significantly increased the phagocytic activity of intraperitoneal macrophages against *Citrobacter rodentium*.

### 3.6. Ex Vivo Effects of LJFE on Peritoneal Macrophage Proliferation in Mice

**Figure 7:**
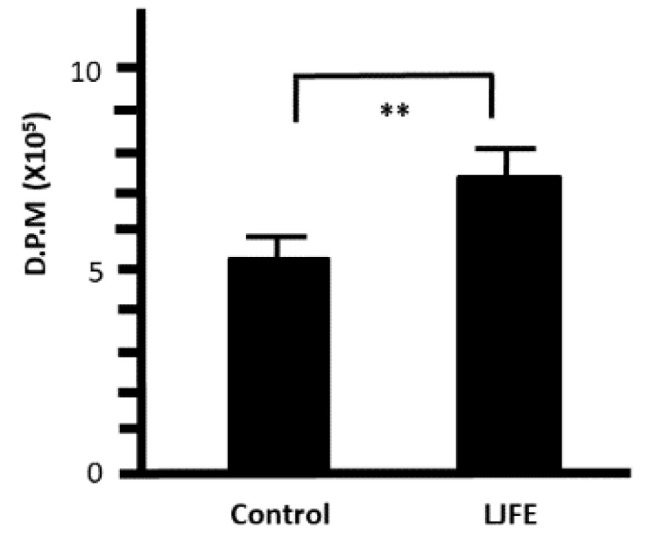
Ex Vivo effect of *Lonicera japonica* flower bud extract (LJFE) on the ^3^H-thymidine uptake of mice intraperitoneal macrophages. Mice were orally administered LJFE (2 g/kg) for seven days, and the ^3^H-thymidine uptake of peritoneal macrophages was measured. Data are presented as mean ± S.D. (*n* = 6). ** *p* < 0.01, *t*-test.

Peritoneal macrophages collected from LJFE-treated mice showed a significantly increased ^3^H-thymidine uptake when compared with those collected from control mice (*p* < 0.01) (Figure 7). From this result, LJFE treatment significantly induced peritoneal macrophage proliferation.

### 3.7. Ex Vivo Effects of LJFE on Cytokine Secretions from Peritoneal Macrophages in Mice

**Figure 8:**
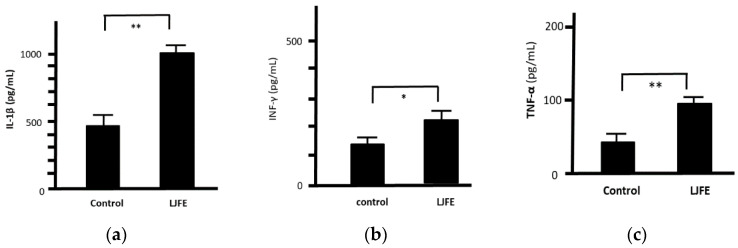
Ex Vivo effect of *Lonicera japonica* flower bud extract (LJFE) on the levels of cytokines secreted from mice intraperitoneal macrophages. Mice were orally administered LJFE (2 g/kg) for seven days, and the levels of interleukin (**a**) (IL)-1β, (**b**) interferon (IFN)-γ, and (**c**) tumor necrosis factor (TNF)-α in the culture supernatant of intraperitoneal cells collected from mice were measured by ELISA. Data are presented as mean ± S.D. (*n* = 6). * *p* < 0.05, ** *p* < 0.01, *t*-test.

The levels of IL-1β, IFN-γ, and TNF-α in the culture supernatant of peritoneal macrophages collected from the LJFE-treated mice were significantly higher than those from the control group (*p* < 0.05, Figure 8). However, we could not confirm any significant difference in IL-6 levels between the two groups (data not shown).

### 3.8. Ex Vivo Effects of CGA on the Phagocytic Activity of Peritoneal Macrophages against Citrobacter rodentium in Mice

**Figure 9:**
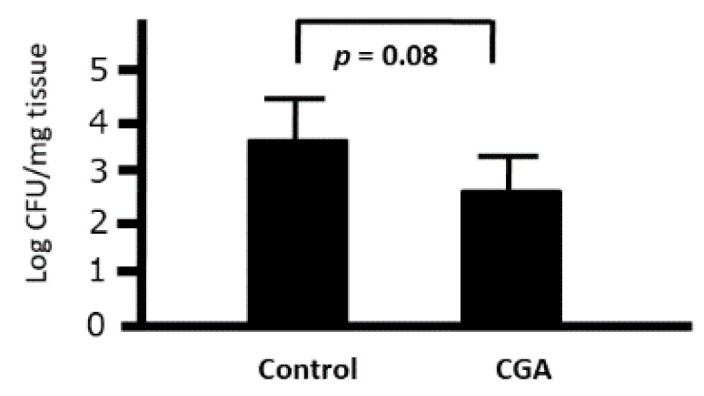
Ex Vivo effects of chrologenic acid (CGA) on the phagocytic activity of intraperitoneal macrophages against *Citrobacter* in mice. Bacterial colonies at 1 h after the start of phagocytosis were demonstrated. Data are presented as mean ± S.D. (*n* = 6). *t*-test.

We investigated the ex vivo effects of CGA on the phagocytic activity of intraperitoneal macrophages. Although the number of bacteria was slightly decreased when cocultured with peritoneal macrophages collected from the CGA-treated group (9.2 mg/kg), we could not find any significant difference between the control and CGA-treated mice (*p* = 0.08) (Figure 9). Therefore, CGA tended to induce the phagocytic activity of intraperitoneal macrophages against *Citrobacter rodentium*.

## 4. Discussion

From the results of this study, we showed that LJFE treatment improved the survival rate; suppressed bacterial colonization in the large intestine, mesenteric lymph nodes, and blood; slightly increased the serum levels of inflammatory cytokines; induced the phagocytic activity of peritoneal macrophages; and elevated inflammatory cytokine secretion from these peritoneal macrophages in *Citrobacter rodentium*-infected mice. Our results support the efficacy of LJFE against *Citrobacter rodentium*-induced gastrointestinal tract infection in an animal model and suggest that the clinical effectiveness of LJFE against infections is not limited to the respiratory system.

Although LJFE did not show direct antibacterial effects against *Citrobacter rodentium*, it likely exerted antimicrobial effects by acting on in vivo immunocompetence. In addition, we speculate that LJFE exhibits antimicrobial effects regardless of the presence of drug-resistant bacteria.

Mice died shortly after oral *Citrobacter rodentium* administration, and we detected bacterial colonization in the mesenteric lymph nodes and blood. Thus, this model can be regarded as a highly invasive infection model, such as enterohemorrhagic *Escherichia coli* O157 in humans. Our results suggest that LJFE administration inhibits bacterial invasion, which is one of the mechanisms underlying the antimicrobial effects of traditional medicines.

In the ex vivo experiments, significant increases in the levels of inflammatory cytokines (IL-1β, IFN-γ, and TNF-α) in both the mouse serum and culture supernatant of intraperitoneal macrophages may explain the effective immunostimulatory mechanism underlying the antibacterial effects of LJFE, such as the repression of bacterial growth in the digestive tract of mice. In addition, considering the slight increase in IL-6 levels, LJFE produced a stronger cell-mediated immunostimulatory effect than humoral immunostimulatory effect. Furthermore, our results showed that the phagocytic activity of intraperitoneal macrophages collected from LJFE-treated mice was increased. This result also supports the possibility that LJFE exerts a strong stimulatory effect on immune cells, such as intraperitoneal macrophages. Finally, the increase in the ^3^H-thymidine uptake of intraperitoneal macrophages in LJFE-treated mice may be involved in promoting the proliferation of immunocompetent cells.

Previous studies have reported several effects of LJF on the immune system. Ethanol extract of LJF showed antibacterial effects against *Staphylococcus aureus* and *Pseudomonas aeruginosa* [13]. Moreover, LJFE showed cytoprotective effects on lipopolysaccharide (LPS)-stimulated RAW264.7 macrophages, and LJFE treatment suppressed LPS-induced increases in nitric oxide (NO), TNF-α, IL-1β, and IL-6 [14]. Furthermore, LJFE could significantly increase splenic lymphocyte proliferation, macrophage phagocytosis, and natural killer cell activity, as well as restore the levels of serum IL-2, TNF-α, and IFN-γ in immunosuppressed mice [14]. Finally, counting T-lymphocyte subsets in the spleen also confirmed the LJFE-induced immunomodulatory effects in immunosuppressed mice [15]. LJFE or CGA isomers demonstrated an anti-inflammatory activity against LPS-induced septic mortality in mice via the suppression of IL-1 receptor-associated kinase-4 [16]. After injection of the egg white, rats in the control group showed obvious toe edema, while LJFE treatment inhibited this toe edema development to some extent. Moreover, in these rats, LJFE showed a certain dose–effect relationship with toe edema, with the strongest inhibitory effects being against inflammation. In a thermoregulation experiment, while the body temperature of rats in the blank group was elevated, it could be lowered in the other groups treated with LJFE. In mice, the severity of ear edema was obvious in the blank control group, as evidenced by significant differences in the thickness between the left and right ears. LJFE treatment inhibited xylene-induced ear edema in mice [17].

Furthermore, previous studies have reported the effects of CGA—an LJFE constituent—on the immune system. CGA activated calcineurin, thereby increasing the phagocytic activity of macrophages. Oral doses of 10 mg/kg, but not 5 mg/kg, have been reported to be effective in mice [18]. However, in our study, this effect was not confirmed, perhaps because our results were obtained in a phagocytosis experiment using live bacteria. However, this effect needs further research in the future. CGA suppressed high-mobility group box 1 expression. It also enhanced toll-like receptor (TLR)-4 expression, and it also inhibited the nuclear factor (NF)-kB and mitogen-activated protein kinase (MAPK) pathways through the activation of the biological defense in mice [19]. CGA strongly inhibited Staphylococcus enterotoxin-induced T-cell proliferation, as well as TNF-α, IL-1β, IL-6, IFN-γ, monocyte chemotactic protein-l, macrophage inflammatory protein (MIP)-lα, and MIP-lβ production by human peripheral blood mononuclear cells [20]. CGA also suppressed LPS-induced NO synthase, NO, IL-1β, IL-6, TNF-α, and MIP-2 expression in RAW264.7 cells by suppressing the NF-κB, c-Jun N-terminal kinase–activator protein-1, and Janus kinase (JAK)2–signal transducer and activator of transcription (STAT)3 pathways [21,22,23].

However, there have been no studies on the effects of LJFE on digestive tract inflammation due to bacterial infection. However, several effects of LJFE on chemical-induced colitis have been demonstrated. LJFE demonstrated protective effects against dextran sulfate-elicited colitis via the Th1/Th17 pathway. LJFE displayed dose-dependent repressive effects against colon shortening, weight loss, and histological damage. LJFE downregulated TNF-α, IL-1β, IL-6, IL-12, IL-17, and IFN-γ [24]. Moreover, hyperoside, lonicerin, and luteolin from LJFE exhibited possible anti-ulcer activities in an animal model of trinitrobenzenesulfonic acid (TNBS) -induced colitis through NF-κB signaling pathway inhibition. These natural compounds significantly reduced the values of specific serum oxidative and proinflammatory markers (superoxide dismutase, myeloperoxidase, malondialdehyde, prostaglandin E2, TNF-α, IL-β, and C-reactive protein) in TNBS -induced colitis [25].

In addition, previous studies have reported the effects of certain crude drugs against Citrobacter sp. Herb supplements (e.g., *Glycyrrhiza glabra*, *Ulmus rubra*, or Triphala formulation) reduced the relative abundance of *Citrobacter freundii* and *Klebsiella pneumoniae* [26]. Methanolic extract of Ajuga parviflora showed an antibacterial activity against *Citrobacter* sp. and *Pseudomonas aeruginosa* [27]. The essential oil of *Artemisia spicigera* represented a powerful antibacterial activity against *Citrobacter amalonaficus*, *Escherichia coli*, *Enterobacter aerogenes*, *Serratia marcescens*, and *Staphylococcus aureus* [28]. Methanolic extract of *Withania somnifera* leaves exhibited a strong activity against *Citrobacter freundii*, *Escherichia coli*, *Pseudomonas aeruginosa*, *Salmonella typhi*, and *Klebsiella pneumoniae* [29]. Methanolic extract of Daucus carota demonstrated an antibacterial activity against *Citrobacter freundii* and *Bacillus cereus* [30]. Methyl protodioscin (MPD) is one of the dominant bioactive components in Dioscoreaceae plants, which are species of yams used for the treatment of chronic inflammatory conditions. MPD protected the colonic mucosa from *Citrobacter rodentium*-elicited inflammation [31]. Although there is no report confirming the presence of MPD in LJFE, it may contain similar constituents, and this should be validated in future studies.

Finally, we evaluated the effects of CGA at a dose equivalent to LJFE (2 g/kg). However, CGA was only slightly effective when compared with LJFE, suggesting that LJFE indeed contains active ingredients other than CGA. LJF has been reported to contain loniceroside, loganin, hydnocarpin, and luteolin, among other components [32,33,34]. Hydnocarpin has been reported to show antibacterial effects against *Staphylococcus aureus* by suppressing biofilm formation [35]. Moreover, loniceroside has been reported to show immunomodulatory effects through its anti-inflammatory activity [36]. Loganin has been reported to alleviate LPS-activated intestinal epithelial inflammation because it modulated the TLR4/NF-κB and JAK/STAT3 signaling pathways [37]. Finally, luteolin isolated from LJF has been reported to suppress inflammatory mediator release because it abrogated the NF-κB and MAPK activation pathways in HMC-1 cells [38]. However, further studies are warranted to confirm the ex vivo effects of these natural compounds on macrophage phagocytosis. Furthermore, although this was not examined in the present study, LJFE may activate neutrophils, lymphocytes, and the complement system, which are biological defense mechanisms against bacteria. Further research on these aspects is warranted as well.

## 5. Conclusions

LJF may be effective in the treatment of *Citrobacter rodentium*-induced digestive tract infections through the upregulation of macrophage phagocytosis and cytokine production. We propose LJFE as an alternate therapeutic agent for human bacterial digestive tract infections.
